# Physiological, biochemical and genetic responses of Caucasian tea (*Camellia sinensis* (L.) Kuntze) genotypes under cold and frost stress

**DOI:** 10.7717/peerj.9787

**Published:** 2020-08-28

**Authors:** Lidiia S. Samarina, Lyudmila S. Malyukova, Alexander M. Efremov, Taisiya A. Simonyan, Alexandra O. Matskiv, Natalia G. Koninskaya, Ruslan S. Rakhmangulov, Maya V. Gvasaliya, Valentina I. Malyarovskaya, Alexey V. Ryndin, Yuriy L. Orlov, Wei Tong, Magda-Viola Hanke

**Affiliations:** 1 Federal Research Centre the “Subtropical Scientific Centre of the Russian Academy of Sciences”, Sochi, Russia; 2 Agrarian and Technological Institute, Peoples’ Friendship University of Russia (RUDN), Moscow, Russia; 3 Novosibirsk State University, Novosibirsk, Russia; 4 State Key Laboratory of Tea Plant Biology and Utilization, Anhui Agricultural University, Hefei, China

**Keywords:** Camellia sinensis, Frost tolerance, Amino acids content, Gene expression, Cations, Osmotic stress, Plant physiology, Climate adaptation

## Abstract

**Background:**

Cold and frost are two serious factors limiting the yield of many crops worldwide, including the tea plant *(Camellia sinensis* (L.) Kuntze). The acclimatization of tea plant from tropical to temperate climate regions resulted in unique germplasm in the North–Western Caucasus with extremely frost-tolerant genotypes.

**Methods:**

The aim of the current research was to evaluate the physiological, biochemical and genetic responses of tolerant and sensitive tea cultivars exposed to cold (0 to +2 °C for 7 days) and frost (−6 to −8 °C for 5 days). Relative water content, cell membranes integrity, pH of the cell sap, water soluble protein, cations, sugars, amino acids were measured under cold and frost. Comparative expression of the following genes ICE1, CBF1, WRKY2, DHN1, DHN2, DHN3, NAC17, NAC26, NAC30, SnRK1.1, SnRK1.2, SnRK1.3, bHLH7, bHLH43, P5CS, LOX1, LOX6, LOX7 were analyzed.

**Results:**

We found elevated protein (by 3–4 times) and cations (potassium, calcium and magnesium) contents in the leaves of both cultivars under cold and frost treatments. Meanwhile, Leu, Met, Val, Thr, Ser were increased under cold and frost, however tolerant cv. Gruzinskii7 showed earlier accumulation of these amino acids. Out of 18 studied genes, 11 were expressed at greater level in the frost- tolerant cultivar comparing with frost-sensitive one*: ICE1, CBF1, WRKY2, DHN2, NAC17, NAC26, SnRK1.1, SnRK1.3, bHLH43, P5CS* and *LOX6*. Positive correlations between certain amino acids namely, Met, Thr, Leu and Ser and studied genes were found. Taken together, the revealed cold responses in Caucasian tea cultivars help better understanding of tea tolerance to low temperature stress and role of revealed metabolites need to be further evaluated in different tea genotypes.

## Introduction

Cold and frost are serious threats to the world agriculture since they cause significant economic damages to the production of many crops, including tea plants. Due to global climate change, the development of new cultivars with increased adaptability to extreme temperatures is becoming an important breeding goal worldwide. The introduction of crops to colder areas could be an efficient strategy to reduce the chemical load of plant protection on commercial plantations, since colder regions are not conducive for the spread of many pests. Efficient breeding for frost tolerance requires a set of informative and stable markers to select the donors of QTLs of tolerance from germplasm. Many studies have led to the development of markers at the morphological, biochemical and genetic levels for selecting tolerant genotypes in several crops (Liu et al., 2017; Xiao et al., 2018; Munne-Bosch, 2014; Zhu et al., 2018).

Tea (*Camellia sinensis* (L.) Kuntze) is a perennial woody crop with a complex response to abiotic stress. Tea plant is cultivated mostly in tropical and subtropical regions of the world, but also in some regions with temperate climate. Commercial plantations of tea in the Caucasus zone consist of a wide range of hybrid genotypes obtained from seeds and plant material imported from China, Japan, India, Sri Lanka and Indonesia. Domestication of the tea plant in the Caucasus occurred within 150 years, during which the tea crop moved from the southern regions of Ozurgetti in Georgia (41°55′27″N 42°00′24″E) to the Northern region in Maykop in Russia (44°36.5858′0″N, 40°6.031′0″E) (Tuov & Ryndin, 2011). Since it is one of the northernmost regions of commercial tea plantations in the world, this germplasm can become a source of frost tolerant genotypes for world breeding and for increasing the world area of commercial tea production. Although tea plantations in this region are smaller than in tea exporting countries, tea production in this region is environmentally safe, since it grows without any application of chemical plant protection. However, in order to conduct an efficient breeding program, it is necessary to develop a reliable set of markers that will help to identify the donors of frost tolerance in collections (Mondal et al., 2004; Mukhopadhyay, Mondal & Chand, 2016).

Tolerance to low temperatures is a quantitative trait and thousands of genes (gene networks) are involved in the cold response in plants (Sanghera et al., 2011). Recently Zheng et al. (2015) showed that the response to cold and frost in tea plant are not completely similar. Moreover, cold tolerance in tea genotypes may depend on the duration of cold or frost exposure (Ban et al., 2017). Furthermore, different mechanisms could provide tolerance to low temperatures in different cultivars. Therefore, studies performed on single cultivars do not give a complete picture of the complex responses to frost in *C*. *sinensis* (L.) Kuntze. The identification of important morphological and physiological mechanisms, as well as the most significant regulatory elements and transcription factors of frost tolerance in the genome is crucial for understanding the comprehensive response of a tea plant to cold and frost.

Some physiological, biochemical and genetic markers of cold tolerance were proposed in certain tea genotypes (Hao et al., 2018). Nevertheless, many mechanisms are still unclear because cold hardiness is a result of a combination of mechanisms involving significant structural, biochemical, and genetic adjustments (Wisniewski, Nassuth & Arora, 2018). These adjustments, which are species-specific (often genotype-specific), are potentially under separate genetic control (Wisniewski, Nassuth & Arora, 2018). It was shown that plants could actively accumulate some amino acids, sugars, and inorganic ions that play important roles during stress response (Hildebrandt et al., 2015; Hildebrandt, 2018; Alberts et al., 1994). The identification of such metabolites and their functions is important for a full understanding of the mechanisms of tea frost tolerance (Li et al., 2019). On the genetic level, *CsICE1* and *CsCBF1* are cold response (COR) genes activated in response and adaptation to low-temperature stress in tea plant (Wang et al., 2012; Yuan et al., 2013; Yin et al., 2016). However it was reported that expression of COR genes is regulated by both the *CBF*-mediated ABA-independent pathway and the *bZIP*-mediated ABA-dependent pathway (Ban et al., 2017). Many transcription factors (*DHN*, *WRKY*, *HD-Zip, LOX, NAC, HSP*) and metabolism genes were showed to be induced in tea in response to cold (Yue et al., 2015; Wu et al., 2015; Wang et al., 2016a; Wang et al., 2016b; Wang et al., 2018a; Wang et al., 2018b; Cui et al., 2018; Chen et al., 2018; Shen et al., 2018; Zhu et al., 2018). Most of these studies used only certain Chinese cultivars, with responses studied at the stage of cold acclimation without subsequent frost induction and responsive mechanisms are not investigated in Caucasian germplasm genotypes.

In the current research, we studied the physiological, biochemical and genetic responses of Caucasian tea cultivars to cold and frost in order to identify the mechanisms underlying their tolerance, and to compare them with previously observed mechanisms in Chinese genotypes and other plants.

## Materials & Methods

### Plant cultivation, cold treatment and sampling

**Figure 1:**
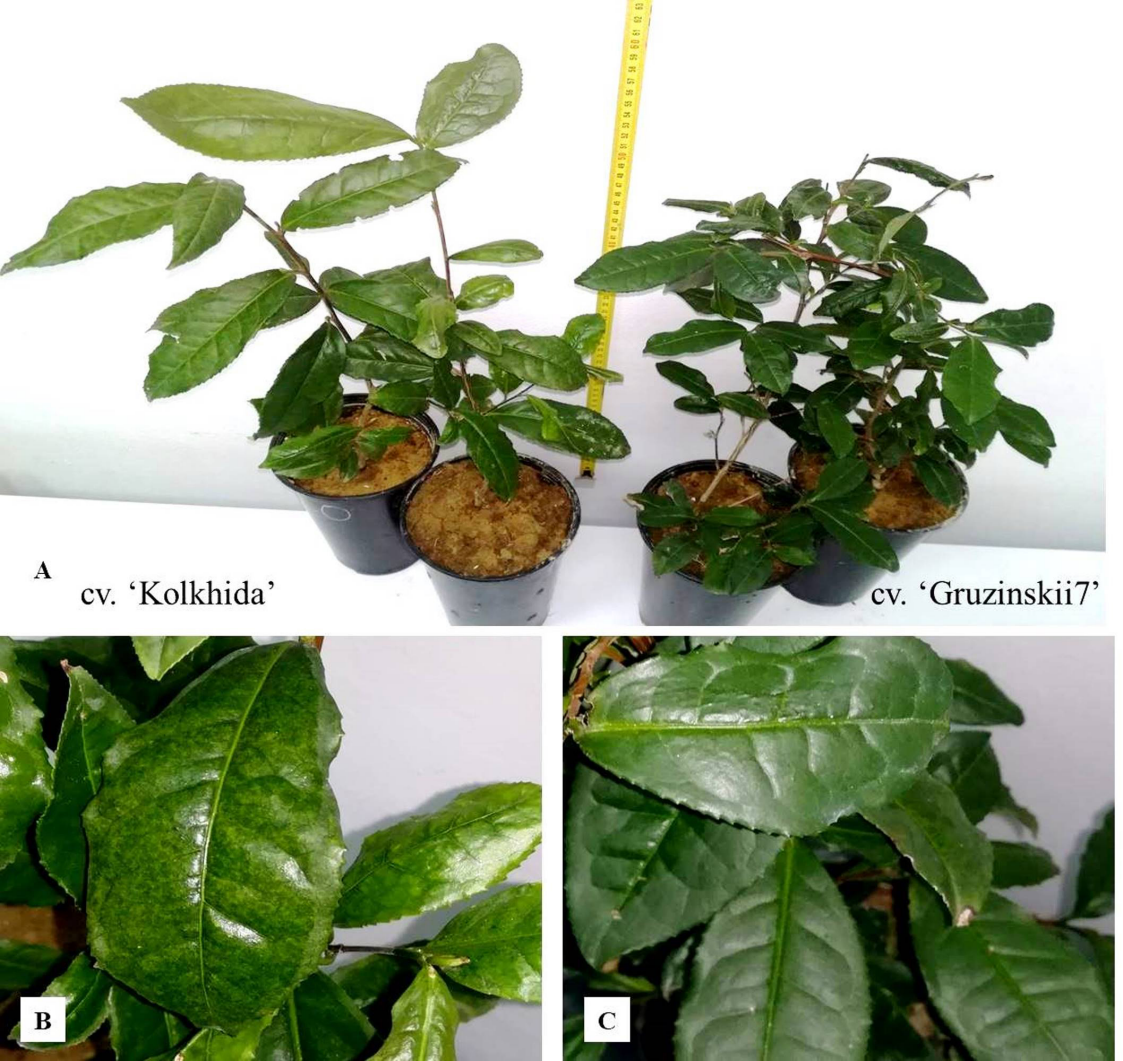
The experiments on tea plants cold and frost induction. (A) Experimental tea plants cvs. ‘Kolkhida’ and ‘Gruzinskii7’ in control conditions; (B) cv. ‘Kolkhida’ after frost treatment; (C) cv. ‘Gruzinskii7’ after frost treatment.

The experiments on cold and frost induction were carried out using two-year-old plants of tea cv. Kolkhida (frost sensitive) and Gruzinskii7 (frost tolerant) (Tuov & Ryndin, 2011; Gvasaliya, 2015) (Fig. 1). Plants were obtained by vegetative propagation of adult tea plants from field collections of the Federal State Budgetary Scientific Institution RRIFSC of two locations: Goitkh (GPS: N44°14′51″E 39°22′33.96″- cultivar Gruzinskii7) and Uch-Dere (GPS: N43°66′89.64″E, 39°63′14.51″- cv. Kolkhida). Both cultivars were shown to survive in −5 °C (cv. Kolkhida) and −15 °C (cv. Gruzinskii7) temperatures. Plants were grown in 2 liter polyethylene pots filled with brown forest acidic soil (pH = 5.0). According to the literature (Hao et al., 2018) cold acclimation of tea plants started when temperature decreased lower than +10. On the other hand, winter comes not immediately after optimum growing period (+18–25 °C) in natural conditions. Therefore medium temperature was selected for the control treatment of plants. Before the cold treatments plants were grown for three months in control conditions with the temperature of +12–14 °C (, with illumination regime of 14 h of light and 10 h of dark, with light intensity of 3000 lux with normal irrigation. Only healthy plants were selected for these experiments. 10 plants of each genotype were included in the study. For each assessed parameter, 2nd, 3rd and 4th mature leaves were used for sampling for each analysis.

Low temperature stress was induced using cold chambers HF-506 (Liebherr, Denmark) as follows: decreasing the temperature by 0–2 °C for 7 days (cold treatment), following decreasing the temperature by −6∼−8 °C for 5 days (frost treatment) to reveal the mechanisms of cold acclimation and frost hardening, respectively. During the treatments, the illumination regime was established as follows: 14 h of light and 10 h of dark every day, with light intensity of 3000 lux.

In order to identify morphological markers of cold tolerance, microstructural parameters of leaves were analyzed by light microscopy using Axio Imager 2 (Carl Zeiss) with the related software. Freshly prepared leaf sections were analyzed in three replicates with 10 fields of view in each.

To determine the (RWC), fresh leaves (FW) were first weighed and then dried at 105 °C for five hours (DW). RWC was calculated according to the formula: $RWC= \left( \frac{ \left( FW-DW \right) }{FW} \right) \ast 100\text{%}$ (Yamasaki & Dillenburg, 1999).

*The cell membranes integrity* was measured with a portable conductivity meter ST300C (Ohaus). 200 mg fresh leaf sample was immersed in 150 ml of deionized water. The measurement of electrical conductivity was done twice: before and after boiling for 60 min at 100 °C. The cell membranes integrity (CMI, %) was calculated using the formula:

\begin{eqnarray*}CMI= \frac{1- \left( \frac{L1}{L2} \right) }{1- \left( \frac{C1}{C2} \right) } \ast 100, \end{eqnarray*}

where L1 and L2 are the conductivity values before and after boiling, C1 and C2 are the relative conductivity of control before and after boiling (averaged over five replicates) (Bajji & Kinet, 2001).

*The pH of the cell sap* was determined potentiometrically on a pH-meter Testo 205. 1 g of fresh leaves was homogenized in 20 ml of distilled water for pH determination using a hydrogen electrode.

*The water-soluble protein* content was determined spectrophotometrically in five replicates according to Bradford protocol (Bradford, 1976; Bonjoch & Tamayo, 2001). The optical density of protein solution was measured at a wavelength of 595 nm on a spectrophotometer USF-01 (Russia).

*Proline* content in leaves (mg g^−1^ fresh leaf mass) was evaluated spectrophotometrically by simplified ninhydrin method (Shihalyeyeva et al., 2014). Absorbance of solution was measured at 520 nm using a spectrophotometer USF-01 (Russia).

*Other amino acids* (arginine, tyrosine, beta-phenylalanine, leucine, methionine, valine, threonine, serine, alpha-alanine, glycine) (mg g^−1^) as well as **sugar content** (mg g^−1^) and** cations** (g g^−1^) were evaluated by capillary electrophoresis on analyzer Kapel-105M (Russia) (Brykalov et al., 2019). Fold-changes of amino acids were counted as the ratio of absolute values cold/control and frost/control.

### Gene expression analysis by qRT-PCR

Total RNA was extracted from the third mature leaf in three biological replicates by the guanidine method with sorption on silica columns, according to the manufacturer’s protocol (Biolabmix, Novosibirsk, Russia). The concentration and quality of RNA was determined on an IMPLEN NPOS 3.1f nano-spectrophotometer and integrity was assessed in a 1% agarose gel. RNA samples were treated with DNaseI; reverse transcription was performed using the MMLV-RT kit (Eurogen). The efficiency of DNaseI treatment and reverse transcription was tested by agarose gel electrophoresis and by qRT-PCR. The results of this verification were evaluated by the presence/absence of a PCR product in RNA samples before and after DNaseI treatment, and by observing the size of PCR fragments in RNA samples before treatment and its cDNA synthesis. Only those samples that confirmed the absence of genomic DNA contamination were included in further analysis of gene expression. This analysis included three groups of samples for each cultivar: the control group - before stress induction, and two experimental groups (cold and frost). To analyze expression differences between two cultivars we focused on the several genes which were previously reported to play important role in abiotic stress-response: *ICE1, CBF1, DHN1, DHN2, DHN3* (Ban et al., 2017), *NAC17, NAC26, NAC30* (Wang et al., 2016b), bHLH7, bHLH43 (Cui et al., 2018), *WRKY2* (Wang et al., 2016a), *LOX1, LOX6, LOX7* (Zhu et al., 2018), *SnRK1.1, SnRK1.2, SnRK1.3* (Yue et al., 2015). *Actin* was taken as a reference gene (Table 1) and results were quantified using a Light Cycler 96 analyzer (Roche). The relative gene expression level was calculated by the Livak & Schmittgen (2001) using following algorithm: 2^−ΔΔ*Cq*^, where:

\begin{eqnarray*}\Delta \Delta Cq=(C{q}_{gene of interest}-C{q}_{internal control})_{treatment}-(C{q}_{gene of interest}-C{q}_{internal control})_{control} \end{eqnarray*}

**Table 1:** Genes and primers for qRT-PCR of tea plant (*Camellia sinensis*.

**Gene**	**Reference**	**Primer sequence 5′–3′**
***Actin***	Hao et al. (2014)	Forward CCA TCA CCA GAA TCC AAG AC Reverse GAA CCC GAA GGC GAA TAG G
*ICE1*	Ban et al. (2017)	Forward ATG TTT TGT AGC CGC AGA C Reverse GCT TTG ATT TGG TCA GGA TG
*CsCBF1*	Ban et al. (2017)	Forward AGA AAT CGG ATG GCT TGT GT Reverse TTG TCG TCT CAG TCG CAG TT
*CsDHN1*	Ban et al. (2017)	Forward ACA CCG ATG AGG TGG AGG TA Reverse AAT CCT CGA ACT TGG GCT CT
*CsDHN2*	Ban et al. (2017)	Forward ACT TAT GGC ACC GGC ACT AC Reverse CTT CCT CCT CCC TCC TTG AC
*CsDHN3*	Ban et al. (2017)	Forward TCC ACA TCG GAG GCC AAA AG Reverse AAC CCT CCT TCC TTG TGC TC
*CsP5CS*	Ban et al. (2017)	Forward AGG CTC ATT GGA CTT GTG ACT Reverse CAT CAG CAT GAC CCA GAA CAG
*CsWRKY2*	Wang et al. (2016a)	Forward GAG ACA GAA ATG AGC AGG GAA AA Reverse TGT ATC GGT GTC AGT TGG GTA GA
*CsNAC17*	Wang et al. (2016b)	Forward CCA AAG AAC AGA GCC ACG Reverse TGG GTA TGA AGG AGT TGG G
*CsNAC26*	Wang et al. (2016b)	Forward ACA AAC TAC GCC ACA ATG C Reverse AGG GAG GGT TCT TTT CAG G
*CsNAC30*	Wang et al. (2016b)	Forward ATT TCA GGG GTT TCA AGC A Reverse CAG AGA ATT CAT TCG CGG
*CsbHLH7*	Cui et al. (2018)	Forward TCA ACG ATC AAC GGA CTT Reverse TCC TCC TCT TCT TCC TCA T
*CsbHLH43*	Cui et al. (2018)	Forward TCT CTG TGC TGC GAA GAC Reverse CCT CCG AGT GTT GCC ATT
*CsSnRK1.1*	Yue et al. (2015)	Forward GTT CAA AAC TCA TCT TCC TCG CT Reverse ATG GTT CTT GTC CAA TCC CAT CT
*CsSnRK1.2*	Yue et al. (2015)	Forward TCT GCT GCT TTA GCT GTG GG Reverse GCT CGA GAC TGT AGG CCA AG
*CsSnRK1.3*	Yue et al. (2015)	Forward TTG GAG TTG CGG TGT CAC TT Reverse CGG GCA CCA TGA GAC AAC T
*CsLOX1*	Zhu et al. (2018)	Forward TCT TGA TTA ATG CCG ATG G Reverse AAA TGC CTC CAA TGG TTC
*CsLOX6*	Zhu et al. (2018)	Forward GAC CCA AGC CTC ACA AAT AG Reverse GCT TCA TTT ATG CTA CTC ACA C
*CsLOX7*	Zhu et al. (2018)	Forward ATT TCT CTT CTC TCA CTC TCA C Reverse GAA CAC CTC TCC ATC ACA CT

### Data analysis, visualization and relationship assessment

All analyses were repeated three times with three to five biological replicates. Statistical analyses were carried out using STATISTICA 6.0 software. One-way ANOVA and Student *t*-test were performed to determine significant differences between the effect of genotype and the respective treatments. For the correlation analysis, the algorithms of nonparametric statistics (Spearman coefficient) were used. The significance of the differences was evaluated by the Fisher test, LSD_05_ and standard deviations from the mean value (Bailey, 1967).

## Results

### Morphological assessment of tea cultivars

**Figure 2:**
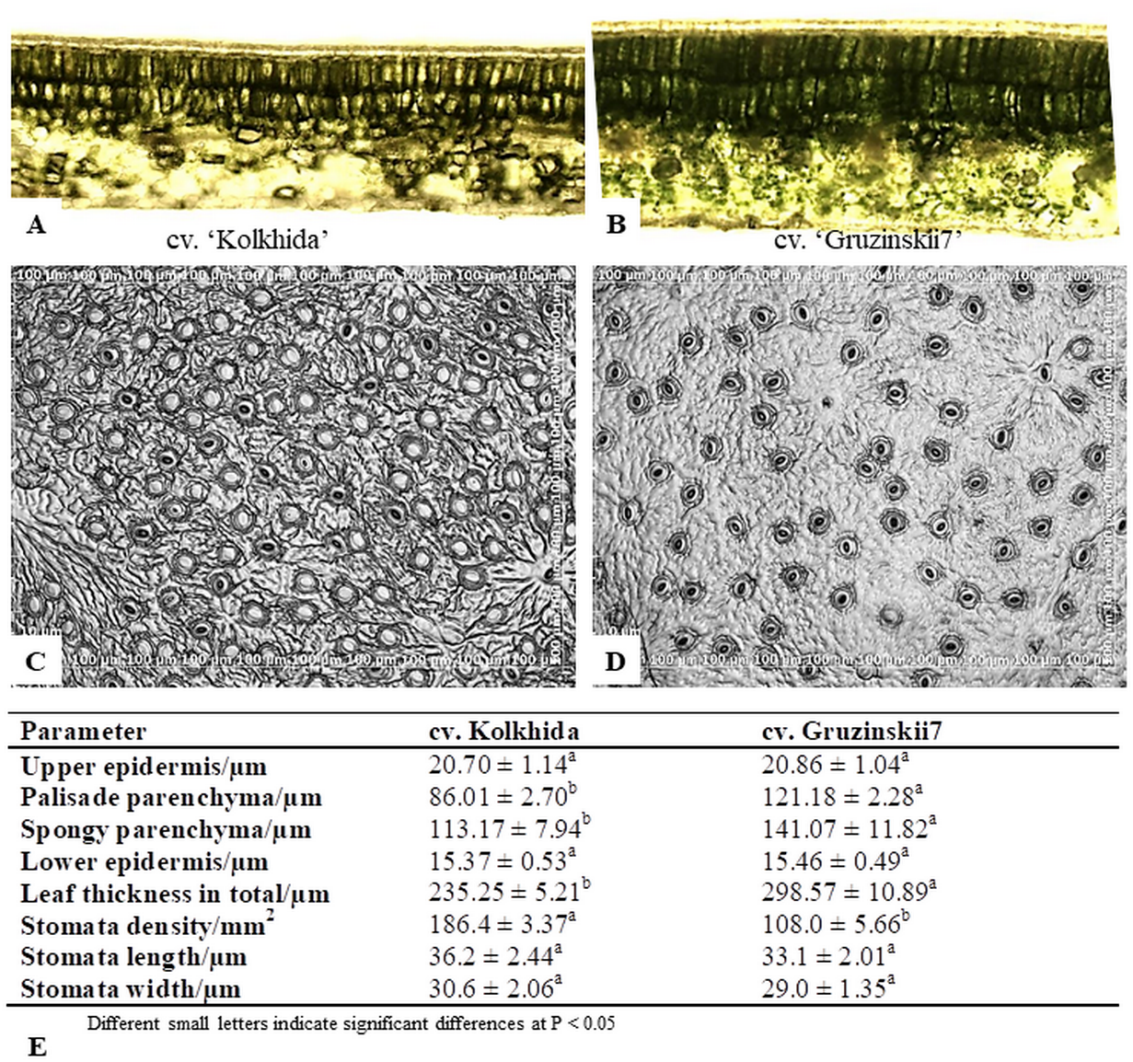
Microstructural evaluations of leaves cross sections and stomata in frost sensitive (‘Kolkhida’) and frost tolerant (‘Gruzinskii7’) tea cultivars (×200). (A) Cross sections of ‘Kolkhida’ leaves; (B) cross sections of ‘Gruzinskii7’ leaves; (C) stomata apparatus of ‘Kolkhida’ leaves; (D) stomata apparatus of ‘Gruzinskii7’ leaves; (E) morphological characteristics of two cultivars.

Morphological tests revealed that the thickness of the upper and lower epidermis were not significantly different between tolerant and sensitive tea plant genotypes. The total thickness of the leaf was ∼298.57 m observed in the tolerant cultivar Gruzinskii7, but only ∼235.25 m in the sensitive cv. Kolkhida (Fig. 2). However, there were significant differences between the two cultivars for thickness of spongy and palisade parenchyma. In cv. Gruzinskii7, both parenchyma layers were significantly thicker compared to the sensitive cv. Kolkhida. Gruzinskii7 was characterized by a lower stomata density as well as smaller stomata size than cv. Kolkhida.

### Physiological response in tea under cold and frost

**Figure 3:**
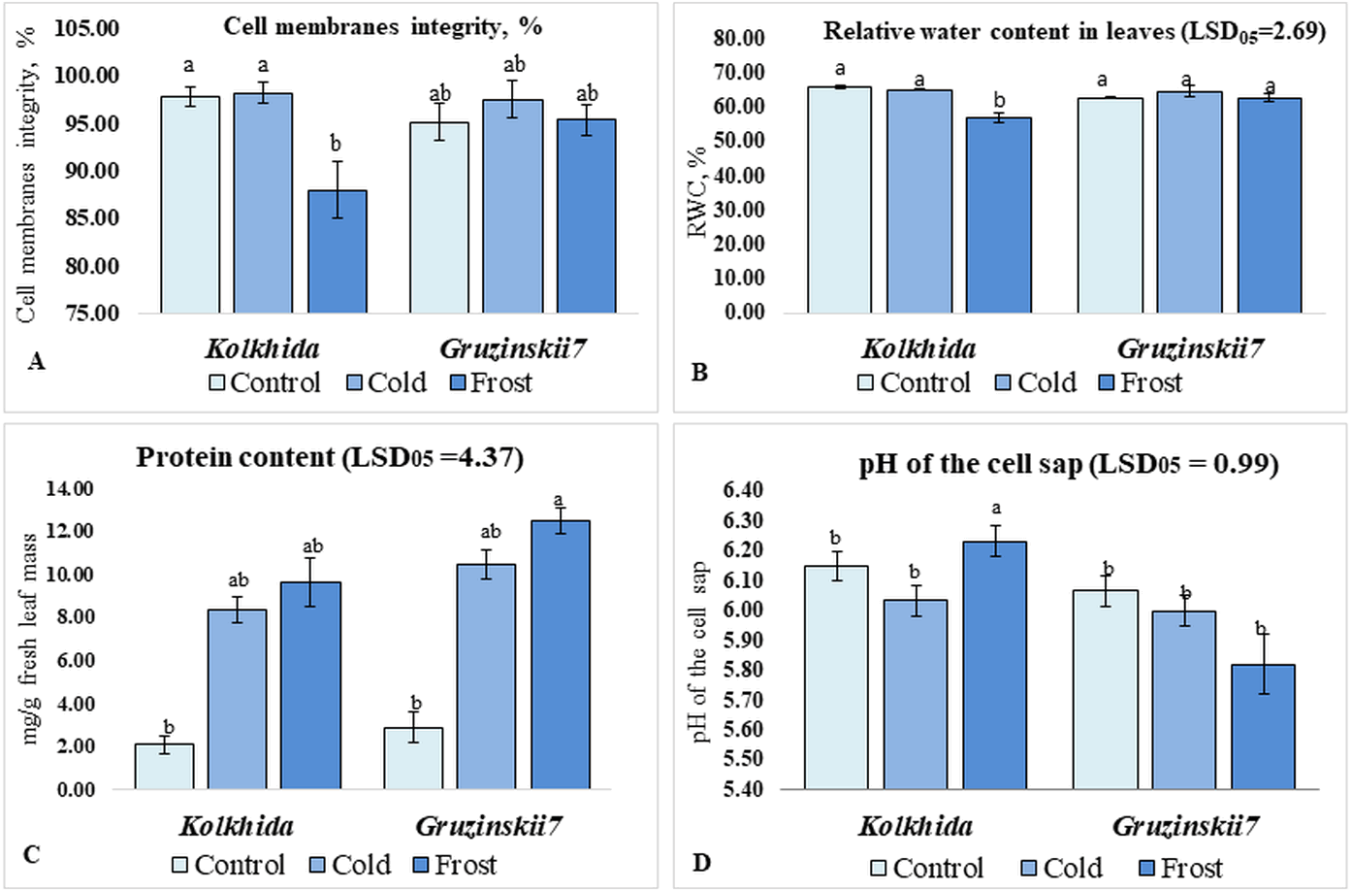
Effect of cold and frost stress on cells. Effect of cold and frost stress on cell membranes integrity (A), relative water content (B), protein content (C) and cell sap pH (D) of leaves in frost-tolerant and frost sensitive tea cultivars. Different lowercase letters indicate significant differences at *P* < 0.05.

Cold treatment did not lead to changes in the CMI, RWC and pH of the cell sap in both tea cultivars. Frost treatment on the other hand resulted in a significant decrease in CMI in cv. Kolkhida, decreased RWC and increase in the pH of the cell sap in cv. Kolkhida, however; no significant changes in these parameters were observed in frost-tolerant cv. Gruzinskii7. Additionally, both cold and frost treatments resulted in increase in the water-soluble protein content by an average of three to four times with no significant differences between two cultivars (Fig. 3).

**Figure 4:**
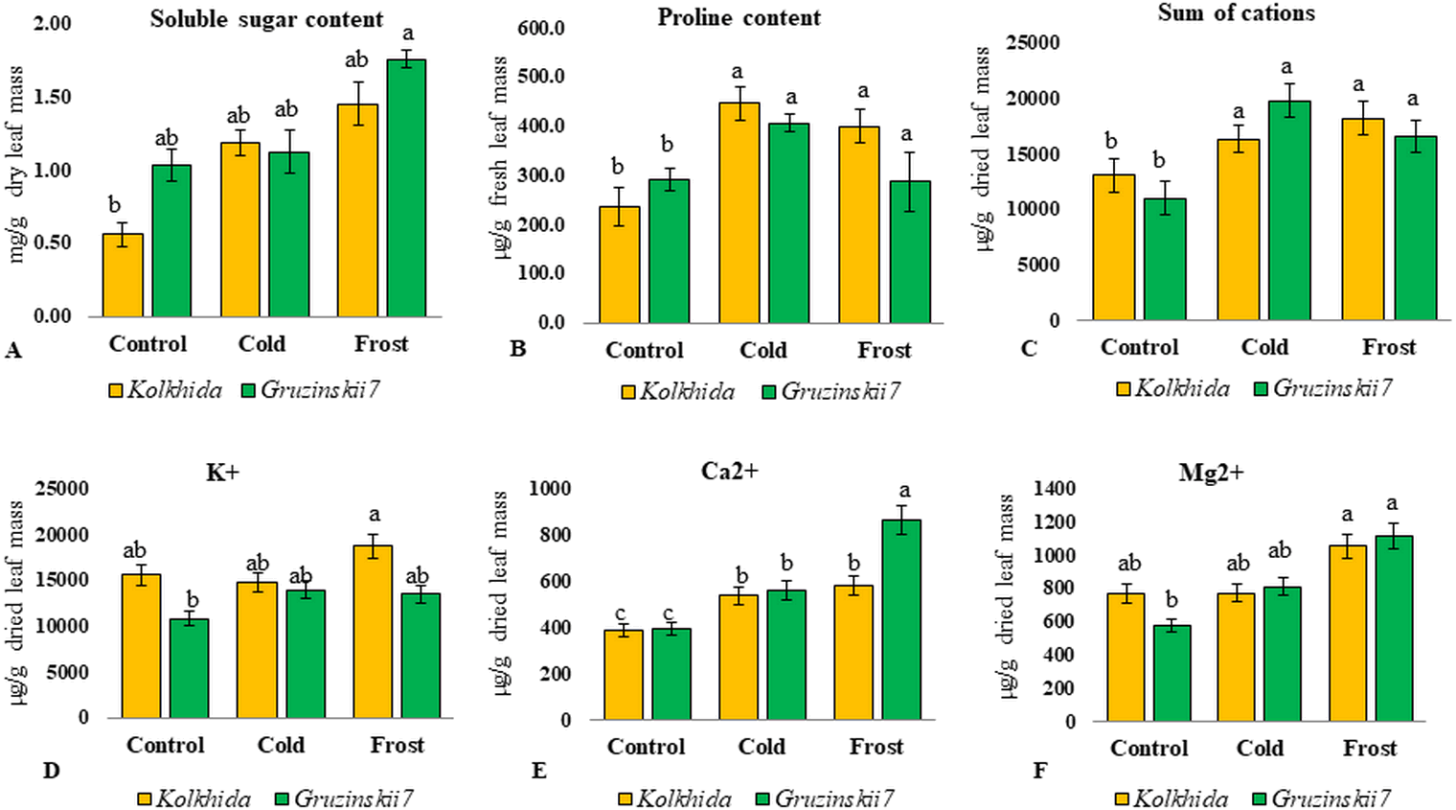
Effect of cold and frost stress on sugars and cations. Effect of cold and frost stress on soluble sugar content (A), proline content (B), Sum of cations (C) and separate cations K^+^ (D), Ca^2+^ (E), Mg^2+^ (F) in leaves in frost-tolerant and frost sensitive tea cultivars. Different lowercase letters indicate significant differences at *P* < 0.05.

Soluble sugars content was elevated during low temperature induction and the highest concentration of sugars was reached in the tolerant cultivar during frost (Fig. 4A). Proline content was also increased significantly in both cultivars under cold induction in both cultivars (Fig. 4B). Sum of cations (NH_4_^+^, Na^+^, K^+^, Mg^2+^, Ca^2+^) in the cell sap was elevated during cold and frost without significant differences between the cultivars (Fig. 4C). Among these five cations, K^+^, Mg^2+^, Ca^2+^ possessed the most pronounced changes during treatments. The highest Ca^2+^ elevation was observed in frost tolerant cultivar under Frost induction (Fig. 4E). The highest K^+^ elevation was observed under frost induction in sensitive cultivar (Fig. 4D). The increase of Mg^2+^ under treatments was not genotype-specific (Fig. 4F).

**Figure 5:**
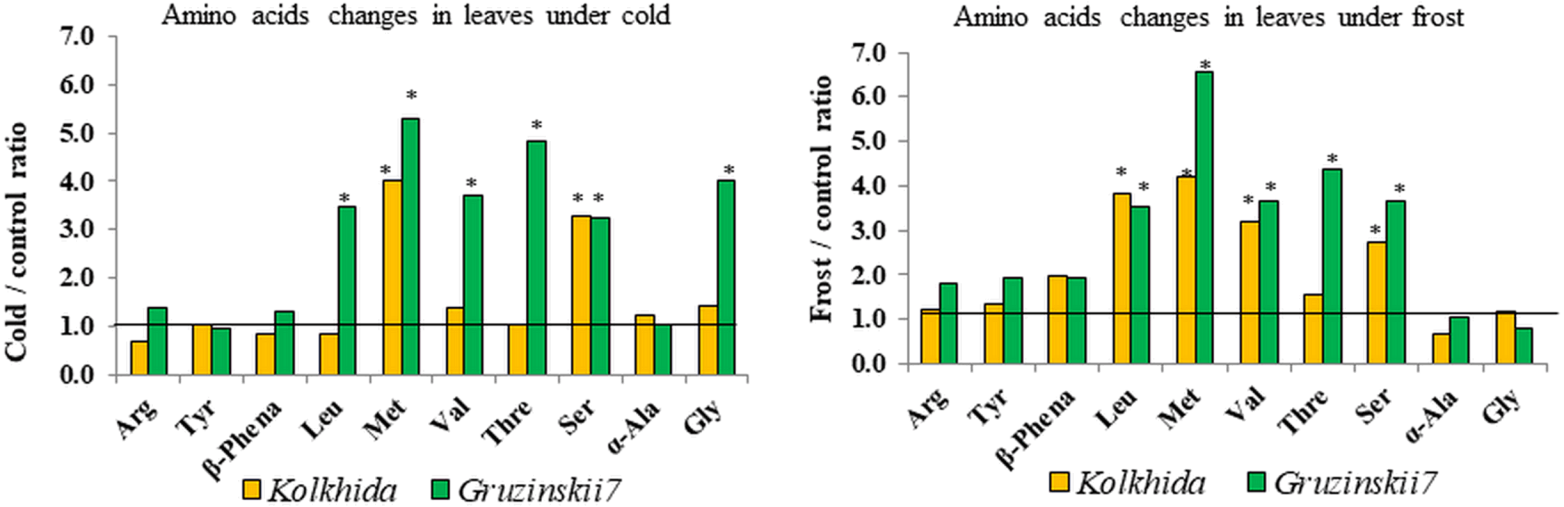
Fold-change of amino acid content in leaf under low temperature stress in tolerant and sensitive tea cultivars (asterisks show significant differences at *P* < 0.05). Threshold shows the level of AA in control group before stress induction.

Due to cold exposure, six amino acids contents increased in comparison to the control (before stress induction) in cv. Gruzinskii7 as follows: serine: 3.24 folds, leucine and valine: 3.5 and 3.7 folds, respectively, glycine: 4.0 folds, threonine: 4.8 folds, and methionine: 5.3 folds. In the sensitive cv. Kolkhida cold exposure led to an increase only in two amino acids as follows: serine by 3.3 folds and methionine by 4.0 folds (Fig. 5).

Similarly, five amino acids increased due to frost induction compared to the control in cv. Gruzinskii7 as follows: leucine, valine, serine: 3.6–3.7 folds, threonine: 4.4 folds, and methionine: 6.55 folds. In sensitive cv. Kolkhida frost treatment resulted in increase of four amino acids: serine: 2.8 folds, valine: 3.2 folds, leucine: 3.8 folds and methionine: 4.2 folds (Fig. 5).

### Relative gene expression in tea under cold and frost stress

**Figure 6:**
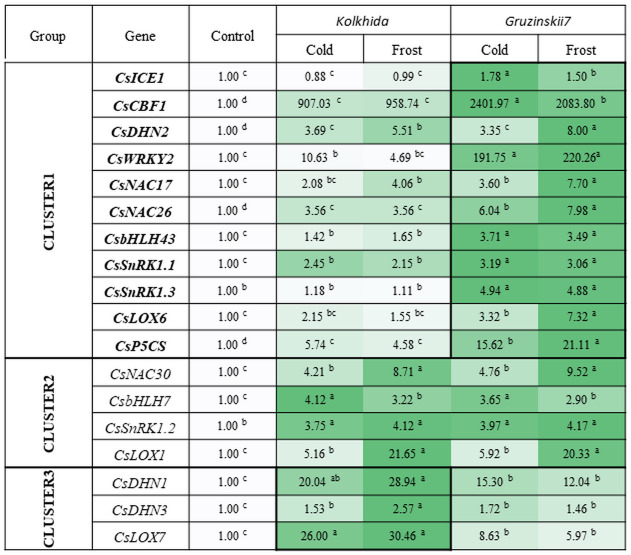
Effect of cold and frost treatments on expression pattern of stress-involved genes in two cultivars of *Camellia sinensis*. Bars represent the mean values of three replicates ±  standard deviation (SD). Different lowercase letters indicate significant differences at *P* < 0.05.

All studied genes divided on three clusters. Cluster 1 included 11 genes with higher expression in the tolerant cultivar. Cluster 2 combined four genes with no difference between two cultivars and Cluster 3—three genes with higher expression in susceptible cultivar under stress conditions (Fig. 6).

The expression level of *ICE1* gene under cold and frost increased by 1.5–1.78 folds in tolerant cultivar Gruzinskii7. Cultivar Kolkhida showed slight increase in expression of this gene with 0.88–1.01 folds under cold and frost. The accumulation of *CBF1* transcripts was dramatically up-regulated by cold. The expression of this gene was genotype-specific and increased by 2400 and 907 folds in cv. Gruzinskii7 and cv. Kolkhida, respectively. After frost treatment, *CBF1* expression slightly decreased in cv. Gruzinskii7.

The expression of *WRKY2* in cv. Gruzinskii7 was extensively induced by cold. The expression of this gene increased by 200 folds, making it the second most important expression level after *CBF1*. In cv. Kolkhida, *WRKY2* increased by 10 and 5 folds under cold and frost treatments, respectively.

Three genes *DHN1*, *DHN2* and *DHN3* exhibited elevated expression with significantly varied expression pattern. The highest expression of the *DHN1* under cold was observed in cv. Kolkhida—by 34 folds. Frost treatment resulted in further increase of *DHN1* expression in cv. Kolkhida but no elevation was observed in cv. Gruzinskii7. The significantly up-regulated expression of *DHN2* and *DHN3* was also observed in both cultivars in response to low-temperature treatment. The accumulation of the transcripts under cold treatment was 4 and 2 folds in *DHN2* and *DHN3* without significant variation between the two genotypes. However, frost treatment lead to the greater accumulation of *DHN2* transcripts in cv. Gruzinskii7 and *DHN3* transcripts in cv. Kolkhida.

Strong induction of *NAC17*, *NAC26* and *NAC30* transcripts was observed in response to cold and frost in both cultivars. Expression of these genes was elevated 4–8 folds (*NAC17*), 6–8 folds (*NAC26*) and 5–10 folds (*NAC30*) in cold and frost, respectively. The tolerant cv. Gruzinskii7 showed greater level of *NAC17* and *NAC26* expression.

The expression of *SnRK1.1, SnRK1.2* and *SnRK1.3* genes was up-regulated and varied in two cultivars under low-temperature treatment. Transcripts of these genes accumulated 2 –5 folds during cold and without further accumulation during the frost treatment. Significant differences between the two cultivars were observed in *SnRK1.1* and *SnRK1.3* expression. The greater accumulation of the transcripts was obtained in cv. Gruzinskii7. The expression profile of *SnRK1.3* was not changed in cv. Kolkhida under cold and frost treatment.

Two genes of *bHLH* family were also extensively expressed in response to the low temperature induction. The accumulation of *bHLH7* transcripts increased 3–4 folds under cold and frost, respectively without difference between the two genotypes. *bHLH43* was strongly up-regulated in the tolerant cultivar in response to cold and frost. The accumulation of its transcripts in cv. Gruzinskii7 increased 4 folds comparing with cv. Kolkhida—1.5 folds.

Three studied *LOX* genes were intensively expressed in response to low-temperature treatment and their expression patterns varied significantly. *LOX1* transcripts were accumulated 6 and 21 folds under cold and frost treatment, respectively with no difference between the two cultivars. However, *LOX6* and *LOX7* expression in response to cold was genotype-specific. *LOX6* exhibited gradually increased expression pattern in the frost-tolerant cultivar with 3–7 folds accumulation under cold and frost, respectively. On the other hand, *LOX7* showed higher expression level in cold-sensitive cultivar Kolkhida—26–30 folds elevation comparing with Gruzinskii7 6–9 folds.

The expression of the *P5CS* gene was also significantly induced by cold treatment. Greater level of the transcript accumulation was observed in cv. Gruzinskii7—16 –21 folds comparing with cv. Kolkhida—5–6 folds after cold and frost treatment, respectively.

### Correlations of tea plant responses to low temperature stress

Positive correlations were observed between *WRKY2* and *ICE1* (*r* = 0.77). Strong correlations were observed between *WRKY2* (*r* = 0.71) and threonine, as well as between *ICE1* and threonine (*r* = 0.78).*CBF1* correlated positively with methionine (*r* = 0.79) and serine ( *r* = 0.74) but negatively with RWC (*r* =  − 0.85). The *P5CS* gene correlated with methionine (*r* = 0.71). A positive correlation was also observed between the pH of the cell sap and leucine (*r* = 0.86) (Table 2).

**Table 2:** Correlation relationships between physiological and molecular responses to low- temperature stress in tea plant.

**Parameter A**	**Parameter B**	**Spearman’s correlation**	**t(N-2)**	*P*-level
***WRKY2***	*ICE1*	0.766	2.923	0.027
	Thr	0.714	2.500	0.047
	Met	0.786	3.111	0.021
***CBF1***	Ser	0.738	2.680	0.037
	RWC	−0.857	−4.076	0.007
***ICE1***	Thr	0.778	3.038	0.045
***PSCS***	Met	0.714	2.500	0.023
**Met**	Ser	0.810	3.378	0.047
**pH of the cell sap**	Leu	0.857	4.076	0.001

## Discussion

There are three phases of plant response to low temperature stress (Hao et al., 2018): the first is acclimation, which occurs at low positive temperatures. The second is hardening, during which the maximum possible degree of frost tolerance is achieved by the plant, and the third phase is the recovery of the plant after stress. We studied the cold acclimation (0 to 2 °C for 7 days) and frost hardening (−6 to −8 °C for 5 days) responses of tea plant using tolerant and sensitive cultivars, in an attempt to reveal the difference in response in two cultivars under cold and frost at the metabolic and genetic levels. In the current study, we used the frost tolerant Caucasian tea genotype Gruzinskii7 to try to identify the molecular mechanisms underlying strong frost tolerance. Specifically, we studied the roles played by early recognition of cold stress (Ban et al., 2017; Hao et al., 2018; Yue et al., 2015), and what specific metabolic pathways, morphological traits (Hirayama & Shinozaki, 2010), and putative genes could be involved in frost tolerance.

Our working hypothesis was that increased cold tolerance of certain tea genotypes could be due to several factors, while fewer factors are triggered in response to cold stress in sensitive cultivars. At the morphological level, greater thickness of parenchyma as well as small size and low density of stomata were the traits found to be specific to frost tolerant cv. Gruzinskii7. These traits are strong indications that cv. Gruzinskii7 recognizes and reacts to low-temperature stress early at the metabolic level.

Since low-temperatures contributes to osmotic stress, due to decrease in the water potential of plant tissues (Crisp et al., 2016), we assessed RWC in leaves as informative indicators of plant response. Frost treatment resulted in decreased RWC in sensitive cv. Kolkhida but not in cv. Gruzinskii7. The adjustment of water potential in the tolerant cultivar could be associated with the structural features of its stomatal apparatus, along with biochemical and molecular regulation. Cell membrane integrity was assessed as another physiological indicator to explain the level of damage caused by stress factor (Ban et al., 2017; Wang et al., 2013; Yue et al., 2015). Our results on CMI are consistent with our data on RWC which confirms reliability of these assessments. Furthermore, frost treatment resulted in a pH shift from acidity to alkalinity in sensitive cv. Kolkhida. These results correspond with our data on the RWC and CMI and consistent with other studies where osmotic stress caused alkalization of cell sap (Netting, 2000; Geilfus, 2017). Therefore, the pH of cell sap could be an efficient marker of ion exchange under low temperature stress.

The content of cations (calcium, magnesium and potassium) increased under low temperature with calcium having the highest elevation in the tolerant cultivar. Changes in the concentration of cations and pH of the cell sap inevitably affect cellular metabolism, enzyme activity, and turgor (Melekhov & Anev, 1991). Calcium was shown to be one of the most important intracellular mediators, which is necessary for a number of basic physiological processes (movement of the cytoplasm, stomatal apparatus, mitosis, growth, hormonal response, etc.) and its concentration is strictly controlled at about 0.1 μ M (Medvedev & Markova, 1990). Entering cells through potential-dependent channels of membranes, Ca^2+^ ions can act as a bioelectric mediator and improve acclimation of higher plants to low temperature stress, so called calcium signal (Vian et al., 1996). Other studies also reported that the calcium signal is a trigger of the cold acclimation process in *Arabidopsis thaliana* (Tähtiharju et al., 1997).

Our results on soluble protein content are consistent with the results obtained in other species such as *O. sativa* and *A. thaliana* in response to cold stress (Karimzadeh et al., 2006; Hildebrandt, 2018). It is also in consistence with the other study reported that tolerant plants showed enhanced levels of proteins under stress conditions, which contribute to maintenance of fully acclimated state (Kosová et al., 2018).

During cold acclimation, six amino acids accumulated in tolerant genotype and only two in sensitive genotype. Frost treatment increased the total amount of soluble amino acids in both tea cultivars, which is consistent with the results of other studies (Kiet, Nose & Zheng, 2016; Hildebrandt, 2018). Accumulation of amino acids was more intense in cv. Gruzinskii7 and possibly made it more tolerant to the subsequent frost treatment. On the other hand, sensitive cv. Kolkhida delayed accumulation of amino acids till exposure to the frost treatment. Some studies also showed the effect of the cultivar with significant differences in the amino acids content under phosphorus deficiency stress (Santosh et al., 2018). We observed an increase in levels of Met, Thr, Val, Leu, Ser, Gly in tea plant under low-temperature stress. Our results are consistent with Hildebrandt (2018) observed an accumulation of glycine, serine, threonine, valine in response to cold stress in *A. thaliana*. Of the 10 amino acids included in our study, the content of protein-bounded amino acids, such as valine, leucine, tyrosine, methionine, increased largely in response to low temperature stress. Although certain protein-bounded amino acids increased in tea under cold and frost, we assumed that their synthesis occurred *de novo*. This assumption is due to our results on the water-soluble protein content, which was increased, and hence active proteolysis not observed under cold and frost conditions in tea. This conclusion is consistent with reports where cold stress did not result in protein degradation unlike other abiotic stresses; nevertheless, several protein-bounded amino acids accumulated under cold in Arabidopsis Hildebrandt (2018).

Met, Thr, Val, Leu, Ser, Gly were earlier reported to play important roles in plant abiotic stresses. Aspartate-derived amino-acids Thr and Met are conjugated with metabolism of branched-chain amino acids Val and Leu which through Ile activate jasmonic acid signaling which is crucial for promoting plants resistance to biotic and abiotic stresses (Jander & Joshi, 2010; Binder, 2010). We observed the highest increase of the sulfur-containing amino acid Met (Binder, 2010). Met is a component of S-adenosylmethionine (AdoMet) with a principal physiological function of sustaining various methylation reactions (Cheng et al., 2003), which could be important for cold-response. AdoMet is also a key element in the regulation of the synthesis of the aspartate-derived amino acids and activates threonine synthase that links with JA signaling through Thr and Ile pathways (Zeh et al., 2001; Ravane et al., 2004). Gly and Ser were shown to participate in joint metabolic pathway with Thr and play important role in plants responses to abiotic stresses (Khan et al., 2017). Ser is an important intermediate in various metabolic pathways in plant metabolism, the one-carbon metabolism and the synthesis of amino acids, such as Gly, Met, Cys, and Trp participating in shikimate way (Tzin & Galili, 2010). In addition, Gly as the component of glycine-rich proteins known to be involved in the regulation of diverse steps in RNA post-transcriptional processing, including splicing and polyadenylation, which are believed to play a crucial role in responses to a variety of detrimental conditions (Czolpinska & Rurek, 2018). Thus, our results confirm the crucial roles played by these six amino acids in tea plant responses to low temperature stress. It is therefore imperative for future work to focus on their metabolism in tea plant under cold conditions.

Further, we studied the expression level of cold -responsive genes in Caucasian tea cultivars, previously suggested to play important roles in cold response of tea plant. *ICE1*—INDUCER OF CBF EXPRESSION 1—is a member of *bHLH* gene family involved in abiotic stress responses in plants (Cui et al., 2018). Previous studies reported no accumulation of *ICE1* transcripts under cold (Ban et al., 2017), and that cold stress (4 °C) did not induce *CsICE1* expression but freezing (−5°C) did (Ding, Ma & Wang, 2016). Our results are not consistent with these findings because we observed elevated *ICE1* expression level at 0–2  °C in frost-tolerant cv. Gruzinskii7. These contradictions are interesting considering the previous reports that *CBF* genes expression begins at 4 ° C and induced by *ICE1* (Wang et al., 2012). However, *ICE1* is more responsible for basic frost tolerance of plants (Zhao et al., 2016). Other two genes of *bHLH* gene family *bHLH7* and *bHLH43* were induced by cold in both our cultivars. These two genes were previously suspected to be involved in abiotic stress responses (heat and drought) (Cui et al., 2018) and in our study we also found its involvement to cold response. According to these researchers *CsbHLH43* was gradually upregulated under cold stress and reached the highest level at 24 h, but *CsbHLH7* was downregulated. Our results showed upregulation of the both genes. So we supposed these genes are interesting for further evaluation in different tea genotypes in response to cold and frost.

Our results confirm the importance of *CBF*—dependent cold response in both tea genotypes. We found that *CBF1* expression was induced by cold and by frost in both cultivars, but much higher it was in cv. Gruzinskii7. We also found high *CBF1* expression during cold acclimation and frost hardening in tea and this is consistent with previous studies that reported the accumulation of *CBF* transcripts only 15 min after cold induction and an increase during subsequent frost (Hua, 2016; Ban et al., 2017).

An important role in the response to cold stress is played by a group of *DHNs* genes encoding dehydrin proteins, which act as cryo protectors, molecular chaperones, and antioxidants (Ban et al., 2017). *DHNs* gene transcription correlates with increased cold tolerance along with *CBF* genes (Paul & Kumar, 2013; Li et al., 2016). In our study, although the expression of the *DHN1, DHN2* and *DHN3* increased significantly, but only *DHN2* transcripts were accumulated at greater level in the tolerant cultivar. This result is not consistent with Li et al. (2016) who reported that *DHN* s expression is greater in frost-tolerant tea genotypes and *DHN1* can be used as marker of frost tolerance. We suppose the additional study with more cultivars is necessary to check this postulation.

Our results also suggest that *CsWRKY2* is crucial since its expression highly increased in tolerant cv. Gruzinskii7 in response to cold and frost. *WRKY* transcription factors play an important role in regulation of plant response to low and high temperatures and to drought stress. A new gene of this family *CsWRKY2* was recently found in tea plant, and its expression increased under cold stress (4 °C) (Wang et al., 2016a; Wang et al., 2016b). *CsWRKY2* plays an important role in signaling pathways with abscisic acid and can be expressed in *CBF*-independent pathway. Results obtained in the current study showed significant differences between tolerant and sensitive cultivars in the expression of this gene under cold and frost.

Recently transcriptome analysis exhibited the expression profiles of *CsNAC* genes in different tea plant cultivars under non-stress conditions. Several *CsNAC* genes, including *CsNAC17* and *CsNAC30* were identified as highly responsive to abiotic stress (Wang et al., 2016b). Our results confirmed the involvement of these genes in low-temperature response. We observed gradually increased expression of *NAC17*, *NAC26* and *NAC30* genes during the cold and frost. In addition, *NAC17* and *NAC26* were greater induced in the frost-tolerant cultivar and can be used as markers for frost-tolerance.

*SnRK1* is a serine/threonine protein kinase whose function is primarily determined by enzyme activity. These genes act as key regulators involved in sugar signaling and involved in the ABA pathway in response to stress stimuli (Jossier et al., 2009). The increase in transcript abundance of *CsSnRK1* during cold indicated that it might facilitate cold acclimation processes in the tea plant (Yue et al., 2015). Our study showed that these three genes were induced by cold. However, *SnRK1.3* expressed at constant level in frost sensitive genotype Kolkhida. Other researchers showed that *SnRK1.2* was induced, *SnRK1.1* was maintained at a relatively constant level, and *SnRK1.3* was sharply suppressed under cold acclimation in tea plant.

The LOX gene family is known to be involved in lipid catabolism for oxylipin synthesis, involved in various defense responses, with ABA salicylic acid (SA) and methyl jasmonate (MeJA) (Liavonchanka & Feussner, 2006). Recently it was reported that *CsLOX1, CsLOX6* and *CsLOX7* transcripts were induced to high accumulation levels in response to 4 °C cold stress and their expression levels were highest at 9, 6 and 12 h, respectively (Zhu et al., 2018). Our results confirmed the involvement of these three genes in cold response. Moreover, we showed that accumulation of these transcripts is greater under cold but not frost treatment. We revealed highly elevated accumulation of LOX1 transcripts in the both tea cultivars. Other two genes were characterized by genotype-dependent expression pathway. LOX6 was mostly induced in tolerant cultivar only, but LOX7 was mostly induced in sensitive cultivar in response to cold. This can be an interesting finding for further studies.

A gene, P5C synthetase (*P5CS*) was involved in proline biosynthesis (Szekely et al., 2008). We observed the highest expression of *P5CS* in the frost tolerant tea genotype cv. Gruzinskii7. Although Ban et al. (2017) also showed an increase in expression of this gene in tea plant under cold, there were however, no clear differences between tolerant and sensitive cultivars. The physiological evaluation of proline content done in the current study also did not find clear differences between two cultivars. Furthermore, there were positive correlations between certain amino acids namely, Met, Thr, Leu and Ser and studied genes that confirm the important roles of these amino acids in tea frost response mechanisms. In conclusion, our results suggest that of the two stress treatments studied, the most informative (diagnostic) stage for selection of frost-tolerant genotypes is the cold acclimation phase. This is consistent with other reported studies (Ban et al., 2017; Hao et al., 2018).

## Conclusions

To conclude, the key findings of the current research are:

1. At the morphological level, greater thickness of parenchyma as well as small size and low density of stomata were the traits found to be specific to frost tolerant cv. Gruzinskii7. These traits are strong indications that cv. Gruzinskii7 recognizes and reacts to low-temperature stress early at the metabolic level.
2. Amino acids Leu, Met, Val, Thr, Ser, Gly are important in tea frost tolerance and positive correlations observed between certain amino acids namely, Met, Thr, Leu and Ser and studied genes.
3. Cations (potassium, calcium and magnesium) content increased under low temperature and can be important mechanism of frost tolerance through stabilizing the cell turgor and ion metabolism.
4. Out of 18 studied genes, 11 were expressed at the higher level in frost tolerant cultivar and can be supposed as markers for frost tolerance: *ICE1*, *CBF1*, *WRKY2, DHN2, NAC17, NAC26, SnRK1.1, SnRK1.3, bHLH43, P5CS* and *LOX6*. The highest expression in response to low temperature was observed in *CBF1* and *WRKY2* genes*.*

These findings will be useful for better understanding of tea tolerance to low temperature stress and to evaluate the reproducibility of frost-tolerance markers in different tea genotypes.

##  Supplemental Information

10.7717/peerj.9787/supp-1 Supplemental Information 1 Raw data from field experiments Data on tea plants in cold, frost and control conditions.

## Additional Information and Declarations

### Data Availability

The following information was supplied regarding data availability: The raw measurements of amino acids content in tea plant leafs are available in the Supplementary Files.

## References

[ref-1] Alberts B, Bray D, Lewis J, Raff M, Roberts K (1994). Molecular biology of the cell.

[ref-2] Bailey NTJ (1967). Mathematical Approach to Biology and Medicine.

[ref-3] Bajji M, Kinet J-M (2001). The use of the electrolyte leakage method for assessing cell membrane stability as a water stress tolerance test in durum wheat. Plant Growth Regulation.

[ref-4] Ban Q, Wang X, Pan C, Wang Y, Kong L, Jiang H, Xu Y, Wang W, Pan Y, Li Y (2017). Comparative analysis of the response and gene regulation in cold resistant and sensitive tea plants. PLOS ONE.

[ref-5] Binder S (2010). Branched-Chain Amino Acid Metabolism in Arabidopsis thaliana. The Arabidopsis Book.

[ref-6] Bonjoch NP, Tamayo PR, Reigosa MJ (2001). Protein content quantification by Bradford method. Handbook of plant ecophysiology Techniques, Roger.

[ref-7] Bradford MM (1976). A rapid and sensitive method for the quantitation of microgram quantities of protein utilizing the principle of protein-dye binding. Analytical Biochemistry.

[ref-8] Brykalov AV, Yakub Yu F, Shanaeva EA, Belik EV, Gryadskikh DA (2019). The use of capillary electrophoresis and gas chromatography for the study of biologically active compounds.

[ref-9] Chen J, Gao T, Wan S, Zhang Y, Yang J, Yu Y (2018). Genome-wide identification, classification and expression analysis of the hsp gene superfamily in tea plant (*Camellia sinensis*). International Journal of Molecular Sciences.

[ref-10] Cheng Z, Sattler S, Maeda H, Sakuragi Y, Bryant DA, DellaPenna D (2003). Highly divergent methyltransferases catalyze a conserved reaction in tocopherol and plastoquinone synthesis in cyanobacteria and photosynthetic eukaryotes. The Plant Cell.

[ref-11] Crisp PA, Ganguly D, Steven RE, Borevitz JO, Pogson BJ (2016). Reconsidering plant memory: intersections between stress recovery, RNA turnover, and epigenetics. Science Adventure.

[ref-12] Cui X, Wang Y-X, Liu Z-W, Wang W-L, Li H, Zhuang J (2018). Transcriptome-wide identification and expression profile analysis of the bHLH family genes in *Camellia sinensis*. Functional & Integrative Genomics.

[ref-13] Czolpinska M, Rurek M (2018). Plant glycine-rich proteins in stress response: an emerging, still prospective story. Frontiers in Plant Science.

[ref-14] Ding ZT, Ma QP, Wang Y (2016). The differences between two tea varieties in their response to natural cold conditions. The Journal of Horticultural Science and Biotechnology.

[ref-15] Geilfus C-M (2017). The pH of the apoplast: dynamic factor with functional impact under stress. Molecular Plant.

[ref-16] Gvasaliya MV (2015). Spontaneous and induced cultivars and forms of tea (*Camellia sinensis* (L.) *Kuntze*) in the humid subtropics of Russia and Georgia: prospects for their cultivation and in vitro conservation.

[ref-17] Hao X, Horvath DP, Chao WS, Yang Y, Wang X, Xiao B (2014). Identification and evaluation of reliable reference genes for quantitative real-time PCR analysis in tea plant (*Camellia sinensis* (L.) O. Kuntze). International Journal of Molecular Sciences.

[ref-18] Hao X, Wang L, Zeng J, Yang Y, Wang X, Han W-Y, Li X, Ahammed G (2018). Response and adaptation mechanisms of tea plant to low-temperature stress. Stress physiology of tea in the face of climate change.

[ref-19] Hildebrandt TM (2018). Synthesis versus degradation: directions of amino acid metabolism during Arabidopsis abiotic stress response. Plant Molecular Biology.

[ref-20] Hildebrandt TM, Nunes NA, Araújo WL, Braun H (2015). Amino acid catabolism in plants. Molecular Plant.

[ref-21] Hirayama T, Shinozaki K (2010). Research on plant abiotic stress responses in the post-genome era: past, present and future. The Plant Journal.

[ref-22] Hua J (2016). De?ning roles of tandemly arrayed *CBF* genes in freezing tolerance with new genome editing tools. New Phytologist.

[ref-23] Jander G, Joshi V (2010). Recent progress in deciphering the biosynthesis of aspartate-derived amino acids in plants. Molecular Plant.

[ref-24] Jossier M, Bouly J-P, Meimoun P, Arjmand A, Lessard P, Hawley S, Grahame HD, Thomas M (2009). SnRK1 (SNF1-related kinase 1) has a central role in sugar and ABA signalling in *Arabidopsis thaliana*. The Plant Journal.

[ref-25] Karimzadeh G, Sharifi-Sirchi GR, Jalali-Javaran M, Dehghani H, Francis D (2006). Soluble proteins induced by low temperature treatment in the leaves of spring and winter wheat cultivars. Pakistan Journal of Botany.

[ref-26] Khan N, Sh Ali, Shahid MA, Kharabian-Masouleh A (2017). Advances in detection of stress tolerance in plants through metabolomics approaches. POJ.

[ref-27] Kiet HY, Nose A, Zheng S-H (2016). Effect of cold stress on root growth, accumulation of soluble proteins and free amino acids of sheath blight-resistant rice genotype 32R. Tropical Agriculture and Development.

[ref-28] Kosová K, Vítámvás P, Urban MO, Práöil IT, Renaut J (2018). Plant abiotic stress proteomics: the major factors determining alterations in cellular proteome. Frontiers in Plant Science.

[ref-29] Li J, Yang Y, Sun K, Chen Y, Chen X, Li X (2019). Exogenous melatonin enhances cold, salt and drought stress tolerance by improving antioxidant defense in tea plant (Camellia sinensis (L.) O. Kuntze). Molecules.

[ref-30] Li YY, Zhou YQ, Xie XF, Shu XT, Deng WW, Jiang CJ (2016). Cloning and transcription analysis of dehydrin gene (*CsDHN*) in tea plant (*Camellia sinensis*). Journal of Agriculture Biotechnology.

[ref-31] Liavonchanka A, Feussner I (2006). Lipoxygenases: occurrence, functions and catalysis. Journal of Plant Physiology/TD.

[ref-32] Liu R, Fang L, Yang T, Zhang X, Hu J, Zhang H, Han W, Hua Z, Hao J, Zong X (2017). Marker-trait association analysis of frost tolerance of 672 worldwide pea (*Pisum sativum* L.) collections. Scientific Reports.

[ref-33] Livak KJ, Schmittgen TD (2001). Analysis of relative gene expression data using realtime quantitative PCR and the 2^−ΔΔ*CT*^ method. Methods.

[ref-34] Medvedev SS, Markova IV (1990). How can the electrical polarity of axial organs regulate plant growth and IAA transport?. Physiologia Plantarum.

[ref-35] Melekhov EI, Anev VN (1991). Reversible exit of K^+^ from the cell as a protective reaction to adverse effects II. Journal of General Biology.

[ref-36] Mondal TK, Bhattacharya A, Laxmikumaran M, Ahuja PS (2004). Recent advances of tea (*Camellia sinensis*) biotechnology. Plant Cell Tissue and Organ Culture.

[ref-37] Mukhopadhyay M, Mondal TK, Chand PK (2016). Biotechnological advances in tea (*Camellia Sinensis* [L.] O. Kuntze): a review. Plant Cell Reports.

[ref-38] Munne-Bosch S (2014). Perennial roots to immortality. Plant Physiology.

[ref-39] Netting AG (2000). pH, abscisic acid and the integration of metabolism in plants under stressed and non-stressed conditions: cellular responses to stress and their implication for plant water relations. Journal of Experimental Botany.

[ref-40] Paul A, Kumar S (2013). Dehydrin2 is a stress-inducible, whereas Dehydrin1 is constitutively expressed but up-regulated gene under varied cues in tea [*Camellia sinensis* (L.) O. Kuntze]. Molecular Biology Reports.

[ref-41] Ravane S, Block MA, Rippert P, Jabrin S, Curien G, Rebeille F, Douce R (2004). Methionine metabolism in plants. The Journal of Biological Chemistry.

[ref-42] Sanghera GS, Wani SH, Hussain W, Singh NB (2011). Engineering cold stress tolerance in crop plants. Current Genomics.

[ref-43] Santosh KC, Liu M, Zhang Q, Fan K, Shi Y, Ruan J (2018). Metabolic changes of amino acids and flavonoids in Tea plants in response to inorganic phosphate limitation. International Journal of Molecular Sciences.

[ref-44] Shen W, Li H, Teng R, Wang Y, Wang W, Zhuang J (2018). Genomic and transcriptomic analyses of HD-Zip family transcription factors and their responses to abiotic stress in tea plant (*Camellia sinensis*). Genomics.

[ref-45] Shihalyeyeva GN, Budnyak AK, Shihalyeyev II, Ivaschenko OL (2014). A modified method for determination of proline in plants. The Journal of V.N. Karazin Kharkiv National University. Series: Biology.

[ref-46] Szekely G, Abraham E, Cseplo A, Rigó G, Zsigmond L, Csiszár J, Ayaydin F, Strizhov N, Jásik J, Schmelzer E, Koncz C, Szabados L (2008). Duplicated P5CS genes of Arabidopsis play distinct roles in stress regulation and developmental control of proline biosynthesis. The Plant Journal.

[ref-47] Tähtiharju S, Sangwan V, Monroy AF, Dhindsa RS, Borg M (1997). The induction of kin genes in cold-acclimating *Arabidopsis thaliana*. Evidence of a role for calcium. Planta.

[ref-48] Tuov MT, Ryndin AV (2011). Perspective tea hybrids in subtropics of the Russian Federation. Subtropical and Ornamental Horticulture.

[ref-49] Tzin V, Galili G (2010). The biosynthetic pathways for shikimate and aromatic amino acids in Arabidopsis thaliana. Arabidopsis Book.

[ref-50] Vian A, Henry-Vian Ch, Schantz R, Ledoigt G, Frachisse J-R, Des-biez M-O, Julien J-L (1996). Is membrane potential involved in calmodulin gene expression after external stimulation in plants?. FEBS Letters.

[ref-51] Wang Y, Jiang CJ, Li YY, Wei CL, Deng WW (2012). *CsICE1* and *CsCBF1*: two transcription factors involved in cold responses in *Camellia sinensis*. Plant Cell Reports.

[ref-52] Wang M, Li Q, Sun K, Chen X, Zhou Q, Li H, Zhang X, Li X (2018b). Involvement of CsCDPK20 and CsCDPK26 in regulation of thermotolerance in tea plant (*Camellia sinensis*). Plant Molecular Biology Reporter.

[ref-53] Wang Y-X, Liu Z-W, Wu Z-J, Li H (2016b). Transcriptome-Wide Identification and Expression Analysis of the NAC Gene Family in Tea Plant [*Camellia sinensis* (L.) O. Kuntze]. PLOS ONE.

[ref-54] Wang Y, Shu Z, Wang W, Jiang X, Li D, Pan J, Li X (2016a). CsWRKY2, a novel WRKY gene from *Camellia sinensis*, is involved in cold and drought stress responses. Biologia Plantarum.

[ref-55] Wang M, Zhang X, Li Q, Chen X, Li X (2018a). Comparative transcriptome analysis to elucidate the enhanced thermotolerance of tea plants (*Camellia sinensis*) treated with exogenous calcium. Planta.

[ref-56] Wang X-C, Zhao Q-Y, Ma C-L, Zhang Z-H, Cao H-L, Kong Y-M, Yue C, Hao X-Y, Chen L, Ma J-Q, Jin J-Q, Li X, Yang Y-J (2013). Global transcriptome profiles of Camellia sinensis during cold acclimation. BMC Genomics.

[ref-57] Wisniewski M, Nassuth A, Arora R (2018). Cold hardiness in trees: a mini-review. Frontiers in Plant Science.

[ref-58] Wu Z, Li X, Liu Z, Li H, Wang Y, Zhuang J (2015). Transcriptome-based discovery of AP2/ERF transcription factors related to temperature stress in tea plant (Camellia sinensis). Functional and Integrative Genomics.

[ref-59] Xiao N, Gao Y, Qian H, Gao Q, Wu Y, Zhang D, Zhang X, Yu L, Li Y, Pan C, Liu G, Zhou C, Jiang M, Huang N, Dai Z, Liang C, Chen Z, Chen J, Li A (2018). Identification of genes related to cold tolerance and a functional allele that confers cold tolerance. Plant Physiology.

[ref-60] Yamasaki S, Dillenburg LR (1999). Measurements of leaf relative water content in *Araucaria angustifolia*. Revista Brasileira de Fisiologia Vegetal.

[ref-61] Yin Y, Ma Q, Zhu Z, Cui Q, Ch Chen, Chen X, Fang W, Li X (2016). Functional analysis of CsCBF3 transcription factor in tea plant (Camellia sinensis) under cold stress. Plant Growth Regulation.

[ref-62] Yuan HY, Zhu XP, Zeng W, Yang HM, Sun N, Xie SX, Cheng L (2013). Isolation and transcription activation analysis of the *CsCBF1* gene from *Camellia sinensis*. Acta Botanica Boreali-Occidentalia Sinica.

[ref-63] Yue C, Cao HL, Wang L, Zhou YH, Huang YT, Hao XY, Wang YC, Wang B, Yang YJ, Wang XC (2015). Effects of cold acclimation on sugar metabolism and sugar-related gene expression in tea plant during the winter season. Plant Molecular Biology.

[ref-64] Zeh M, Casazza AP, Kreft O, Roessner U, Bieberich K, Willmitzer L, Hoefgen R, Hesse H (2001). Antisense inhibition of threonine synthase leads to high methionine content in transgenic potato plants. Plant Physiology.

[ref-65] Zhao C, Zhang Z, Xie S, Si T, Li Y, Zhu JK (2016). Mutational evidence for the critical role of CBF transcription factors in cold acclimation in Arabidopsis. Plant Physiology.

[ref-66] Zheng C, Zhao L, Wang Y, Shen J, Zhang Y, Jia S, Li Y, Ding Z (2015). Integrated RNA-Seq and sRNASeq analysis identifies chilling and freezing responsive key molecular players and pathways in tea plant (*Camellia sinensis*). PLOS ONE.

[ref-67] Zhu J, Wang X, Guo L, Xu Q, Zhao S, Li F, Yan X, Liu S, Wei C (2018). Characterization and alternative splicing profiles of the lipoxygenase gene family in tea plant (*Camellia sinensis*). Plant and Cell Physiology.

